# Antimicrobial Resistance Patterns in Infections Among Patients With Hematologic Malignancies: A Systematic Review

**DOI:** 10.7759/cureus.111932

**Published:** 2026-07-02

**Authors:** Tanveen Kaur Soni, Abhishek Hanumanpratap Singh Kshatri, Aysha Femy, Bhanu Verma, Pranav Verma, Subhasree Dutta, Shreya Bodanam, Lalitha Soumya Johnson, Keerthana R, Jnapika Devarapalli, Akshay V P, Delna NS, Shubhrit Shrivastava, Sangram Keshari Samal

**Affiliations:** 1 Biotechnology, JIS University, Kolkata, IND; 2 Emergency Medicine, Apollo Hospitals, Health City, Visakhapatnam, IND; 3 General Medicine, Batterjee Medical College, Dammam, SAU; 4 Pharmacy, University School of Pharmaceutical Sciences, Rayat Bahra University, Mohali, IND; 5 Pharmacy, Chitkara College of Pharmacy, Chitkara University, Rajpura, IND; 6 Molecular Biology, Bodoland University, Kokrajhar, IND; 7 Internal Medicine, Kamineni Academy of Medical Sciences and Research Centre, Hyderabad, IND; 8 Biotechnology and Microbiology, Dr. Janaki Ammal Campus, Kannur University, Kannur, IND; 9 Pathology, KBN Institute of Medical Sciences, Bengaluru, IND; 10 Internal Medicine, Gayatri Vidya Parishad Institute of Healthcare and Medical Technology, Pataparadesipalem, IND; 11 Biotechnology, Mansarovar Global University, Bhopal, IND; 12 Molecular Biology, BioDeskINDIA Labs, Bhopal, IND; 13 Allied Health Sciences, Al-Azhar Medical College and Super Speciality Hospital, Thodupuzha, IND; 14 Biochemistry, BioDeskINDIA Labs, Bhopal, IND; 15 Biotechnology, Kalinga University Raipur, Naya Raipur, IND

**Keywords:** antimicrobial resistance, bacterial infections, blood cancers, bloodstream infections, hematologic malignancies, hematologic neoplasms, immunocompromised patients, multidrug-resistant organisms, systematic review

## Abstract

Antimicrobial resistance is an important clinical challenge in patients with hematologic malignancies, who are highly susceptible to severe infections due to prolonged neutropenia, disrupted mucosal barriers, intensive chemotherapy, and hematopoietic stem-cell transplantation. This systematic review synthesizes available international evidence on pathogen distribution, antimicrobial resistance patterns, and clinical outcomes in hemato-oncology settings. This systematic review searched PubMed, Scopus, and Google Scholar for English-language studies published from January 2014 to 15 November 2025. Studies reporting microbiologically confirmed infections in patients with hematologic malignancies, with extractable antimicrobial susceptibility or resistance data, were included. Due to substantial clinical, microbiological, methodological, and epidemiological heterogeneity, findings were synthesized qualitatively. Seventeen studies met the inclusion criteria. Gram-negative bacteria predominated across most settings, although pathogen distribution varied by region, age group, infection type, and denominator unit. Extended-spectrum β-lactamase (ESBL)-producing *Enterobacterales*, carbapenem-resistant Gram-negative bacteria, multidrug-resistant Gram-negative organisms, methicillin-resistant *Staphylococcus aureus*, and vancomycin-resistant *Enterococcus* were reported across included studies. Where extractable study-level data were available, ESBL-related estimates ranged from 40% to 78%, while carbapenem-resistance estimates varied widely by organism group and study setting, from low carbapenem resistance among *Enterobacteriaceae* in one pediatric oncology cohort to 38.2% carbapenem-resistant Gram-negative bacteria in an Indian adult febrile neutropenia cohort. Mortality outcomes were heterogeneously reported and were not pooled. In comparative studies, resistant or multidrug-resistant bloodstream infections were associated with worse outcomes, including increased mortality and ICU admission, although causality cannot be inferred from the predominantly observational evidence. Antimicrobial resistance is a clinically important problem in patients with hematologic malignancies, particularly due to resistant Gram-negative pathogens. The findings support strengthened hemato-oncology-specific surveillance, local antibiogram-guided empirical therapy, antimicrobial stewardship, and standardized reporting of resistance phenotypes and clinical outcomes.

## Introduction and background

Infections remain one of the leading causes of morbidity and mortality among patients with hematologic malignancies worldwide. These patients are uniquely vulnerable because both the underlying malignancy and its treatment disrupt multiple layers of host defense [1]. Cytotoxic chemotherapy, radiotherapy, immunosuppressive therapy, monoclonal antibodies, and hematopoietic stem-cell transplantation (HSCT) can impair innate and adaptive immunity. Prolonged neutropenia, mucosal barrier injury, central venous catheter use, repeated hospitalization, and exposure to broad-spectrum antimicrobials further increase the risk of severe bacterial and fungal infections. In this setting, even initially localized infections may progress rapidly to bloodstream infection (BSI), sepsis, septic shock, intensive care unit (ICU) admission, and death if effective antimicrobial therapy is delayed [1,2].

Antimicrobial resistance (AMR) has become a major clinical concern in hemato-oncology practice. Empirical and prophylactic antimicrobial therapy is frequently used in patients with febrile neutropenia to reduce early infectious mortality. However, repeated or prolonged antimicrobial exposure can select for multidrug-resistant (MDR) pathogens [2-4]. This risk is amplified in high-acuity hospital environments, where resistant organisms may spread through catheter-associated infections, gastrointestinal colonization, environmental reservoirs, and healthcare-associated transmission [3,4]. As a result, clinicians treating patients with hematologic malignancies must balance the need for immediate broad-spectrum therapy against the risk of promoting further resistance.

The microbiological profile of infections in hematologic malignancy has changed over time. Gram-positive organisms have historically been important in catheter-associated and mucositis-related infections. However, MDR Gram-negative pathogens are now increasingly recognized as major causes of severe infection in hemato-oncology settings [5]. Extended-spectrum β-lactamase (ESBL)-producing *Escherichia coli* and *Klebsiella pneumoniae*, carbapenem-resistant *Enterobacterales*, MDR *Pseudomonas aeruginosa*, and MDR or extensively drug-resistant *Acinetobacter baumannii* are clinically important because they limit empirical treatment options and may require last-line agents such as colistin, tigecycline, or newer β-lactam/β-lactamase inhibitor combinations. In immunocompromised patients, inappropriate initial therapy can lead to rapid clinical deterioration and poor outcomes [1,2,5].

AMR in this population is not only a microbiological problem but also a clinical and stewardship challenge. Resistance patterns influence empirical antibiotic selection, escalation and de-escalation strategies, infection-control policies, and local antibiogram development. This is particularly relevant in low- and middle-income countries, where high antimicrobial pressure, variable diagnostic access, and differences in infection-control infrastructure may contribute to greater resistance burdens. At the same time, resistance patterns vary substantially across regions, age groups, treatment settings, infection syndromes, and denominator units, making broad conclusions difficult without careful synthesis.

Previous reviews have addressed resistant bacterial infections in patients with hematologic malignancies. However, many prior syntheses were geographically restricted, pathogen-specific, resistance-phenotype-specific, or focused on selected high-risk subgroups such as HSCT recipients or febrile neutropenia cohorts. An updated synthesis with broader geographic coverage and wider AMR scope is therefore needed to clarify the current evidence base, identify recurring resistance patterns, and highlight gaps relevant to surveillance and clinical practice.

The present systematic review was designed to synthesize available evidence on AMR among infections in patients with hematologic malignancies. This review aims to summarize AMR patterns, identify major resistant pathogens, describe associated clinical outcomes, and highlight evidence gaps relevant to surveillance, antimicrobial stewardship, and empirical treatment strategies.

## Review

Materials and methods

Study Design and Registration

This systematic review was conducted in accordance with the Preferred Reporting Items for Systematic Reviews and Meta-Analyses (PRISMA) 2020 guidelines [6]. The review protocol was prospectively registered in PROSPERO (CRD420251001142).

Eligibility Criteria

Using a PEO framework, this review aims to synthesize available evidence on antimicrobial resistance patterns (Exposure) among pathogens causing infections in patients with hematologic malignancies (Population) and to describe associated clinical outcomes, including resistance prevalence, pathogen distribution, and infection-related morbidity and mortality (Outcome) [3].

Inclusion Criteria and Exclusion Criteria

Studies were eligible for inclusion if they reported microbiologically confirmed bacterial or fungal infections occurring in patients diagnosed with hematologic malignancies. Eligible studies were required to provide extractable antimicrobial susceptibility or resistance data for the identified pathogens. Both observational (prospective or retrospective) and experimental study designs were considered for inclusion. Studies were excluded if they did not report antimicrobial resistance data or lacked sufficient microbiological detail to allow interpretation of resistance patterns. Studies focusing exclusively on non-hemato-oncology populations were excluded. Conference abstracts, narrative reviews, editorials, commentaries, case reports, and studies with incomplete or inaccessible data were excluded to ensure data reliability. Publications not available in the English language were excluded due to feasibility constraints in data extraction and interpretation.

Definitions of Antimicrobial Resistance

MDR definitions were recorded as reported by the original studies. Where explicitly defined, MDR generally referred to resistance to at least one antimicrobial agent in three or more antimicrobial classes. However, definitions were not fully uniform across studies, and some studies reported MDROs (multidrug-resistant organisms) using local laboratory or institutional definitions. For this reason, MDR and MDRO values were summarized descriptively and were not pooled. ESBL production and carbapenem resistance were recorded as reported by the original studies. Where available, studies using Clinical and Laboratory Standards Institute (CLSI) or European Committee on Antimicrobial Susceptibility Testing (EUCAST) breakpoints were noted [2].

Information Sources and Search Strategy

Systematic searches were conducted in PubMed, Scopus, and Google Scholar to identify studies reporting antimicrobial resistance patterns among infections in patients with hematologic malignancies. The search covered studies published from January 2014 to 15 November 2025. The original PROSPERO registration specified a planned search end date of 31 December 2024. Before manuscript submission, an updated search was conducted up to 15 November 2025 to capture recently published eligible studies and improve the currency of the review. This extension did not alter the review question, eligibility criteria, outcomes, or synthesis approach. The updated search period has been reported transparently in the manuscript and recorded as a protocol amendment/clarification where applicable.

Controlled vocabulary was adapted according to the database structure. In PubMed, Medical Subject Headings (MeSH) were combined with free-text terms related to hematologic malignancies, antimicrobial resistance, MDROs, and infection. In Scopus, the strategy was adapted using title, abstract, and keyword fields because Scopus does not use MeSH indexing. Google Scholar was used as a supplementary source with simplified free-text combinations because it does not support reproducible database-style controlled vocabulary searching.

Additional studies were identified through reference list screening and by contacting authors when clarification or missing data were required. The PubMed search strategy combined MeSH and free-text terms related to hematologic malignancies and antimicrobial resistance. The complete search string is provided in the appendices.

Google Scholar was searched as a supplementary source to capture additional studies not indexed in PubMed or Scopus. Due to the non-reproducible nature of Google Scholar’s ranking algorithm, the search was restricted to the first 200 results, sorted by relevance. A complete search strategy was documented in the full protocol available in OSF (Open Science Framework) [7].

Study Selection

Two reviewers independently screened titles and abstracts to identify potentially eligible studies. Full texts of shortlisted articles were assessed for inclusion according to predefined criteria. Discrepancies were resolved through discussion or by consulting a third reviewer.

Data Extraction

Data were extracted independently by two reviewers using a standardized data extraction sheet. Extracted variables included (1) study characteristics (author, year, country, design, population), (2) sample size and type of clinical specimens, (3) pathogens identified, (4) reported antimicrobial susceptibility or resistance patterns, and (5) clinical outcomes such as infection-related complications, ICU admission, length of hospital stays, and mortality.

Quality Assessment

Risk of bias was assessed separately for cohort and cross-sectional studies. Cohort studies were evaluated using the Newcastle-Ottawa Scale (NOS), which assesses selection, comparability, and outcome domains, with a maximum score of nine points. Studies scoring ≥7 were categorized as low risk of bias, studies scoring 5-6 as moderate risk of bias, and studies scoring <5 as high risk of bias. Cross-sectional studies were evaluated using a modified NOS adapted for cross-sectional designs, with assessment domains including sample representativeness, sample size, non-response, exposure and outcome ascertainment, confounder control, statistical testing, and data completeness [8].

Among the 13 cohort studies included in the review, five were categorized as low risk of bias and eight as moderate risk of bias. No cohort study was categorized as high risk of bias. Among the four cross-sectional studies, all were categorized as low risk of bias using the modified NOS. Overall, nine studies were classified as low risk of bias and eight as moderate risk of bias.

Data Synthesis

Due to substantial clinical, methodological, microbiological, and epidemiological heterogeneity, quantitative meta-analysis was not performed. The included studies varied by geographic setting, age group, hematologic diagnosis, treatment exposure, HSCT status, infection syndrome, specimen type, pathogen category, antimicrobial susceptibility-testing standard, resistance definition, denominator unit, and clinical outcome reporting.

Resistance data were reported using different units of analysis, including patients, infection episodes, bloodstream infection episodes, isolates, tested isolates, cultures, and specimens. Some studies reported resistance descriptively without extractable numerator-denominator data. Therefore, antimicrobial resistance findings were summarized descriptively at the study level by pathogen group, resistance phenotype, denominator type, and clinical outcome. Values were not pooled, weighted, or treated as directly comparable prevalence estimates.

Clinical outcomes were summarized according to the original definitions used by each study, including overall mortality, infection-related fatality, 30-day mortality, case-fatality, bacteremia-attributable mortality, ICU admission, complications, and treatment response, where reported. Because no pooled meta-analysis was performed, I², Cochran’s Q, funnel plots, and Egger’s test were not applicable. This deviation from the originally planned quantitative synthesis was reported transparently, and a descriptive study-level synthesis was performed instead.

Results

**Figure 1:**
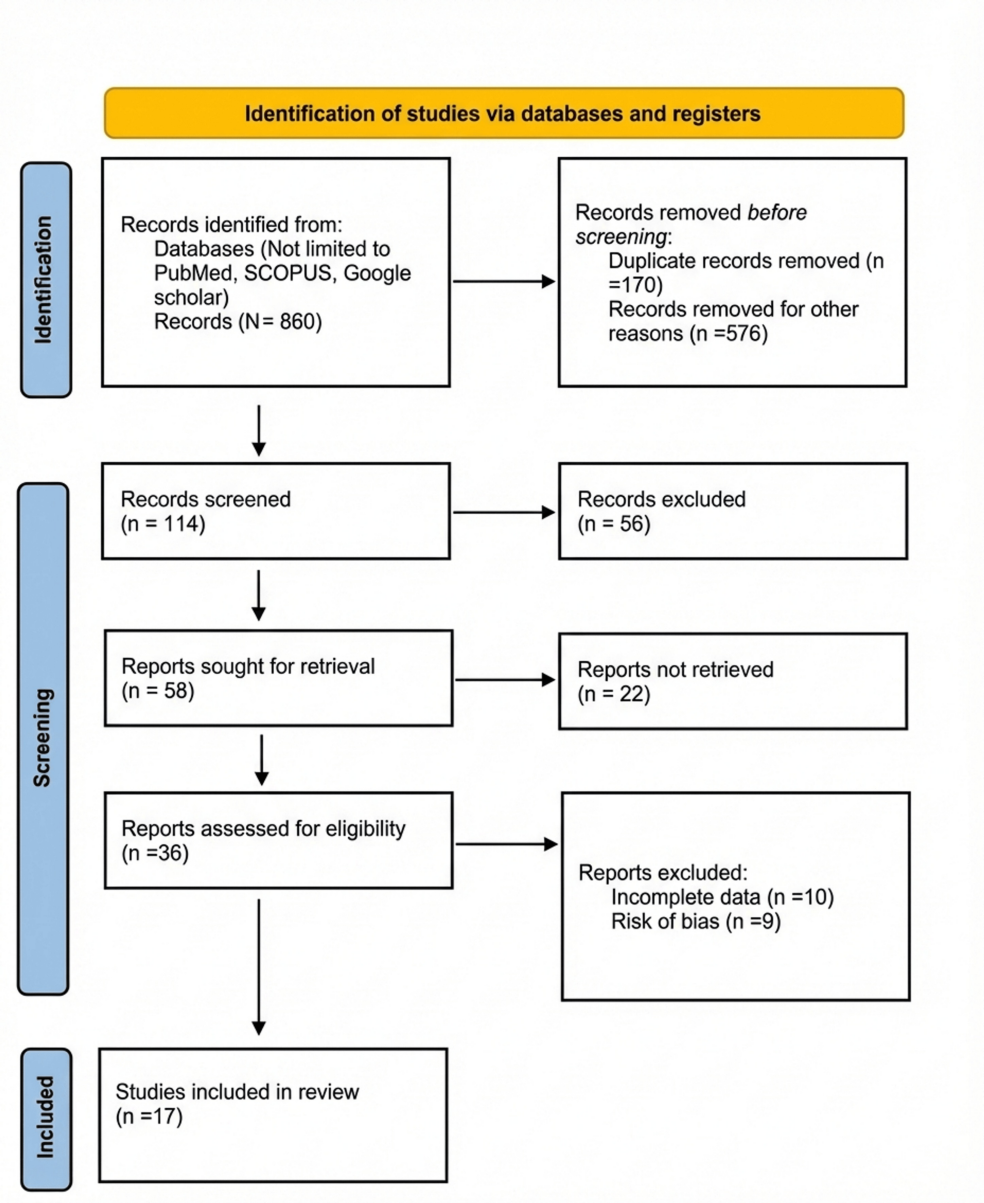
PRISMA flow diagram of the study selection process. PRISMA: Preferred Reporting Items for Systematic Reviews and Meta-Analyses Source: [6]

The initial search identified a total of 860 records. Following duplicate and preliminary exclusion, 114 records were screened. Fifty-eight reports were sought for retrieval, 36 full texts were assessed for eligibility, and 17 studies were included. A PRISMA flow diagram (Figure 1) summarizes the selection process.

Study Characteristics

The 17 included studies encompassed a mixture of retrospective cohorts (n=13) and prospective observational studies (n=4). Four studies focused exclusively on pediatric hemato-oncology cohorts, while the others reported on adults or mixed-age populations. The studies were geographically diverse but unevenly distributed. Asia contributed the largest number of studies, including India, Qatar, Indonesia, Turkey, Japan, Hong Kong, and Palestine. Europe was represented by studies from Poland, Italy, and Germany. Africa was represented by one study from South Africa, and Latin America by one study from Mexico. One study was conducted in Australia and was categorized separately as Oceania/Australia. No eligible study from North America was included. Because antimicrobial resistance patterns vary substantially by region, findings were interpreted descriptively within the regional context rather than pooled across regions (Tables 1, 2).

**Table 1:** Data extracted from studies included in the review. AMR, antimicrobial resistance; BSI, bloodstream infection; ESBL, extended-spectrum β-lactamase; MDR, multidrug-resistant; MDRO, multidrug-resistant organism; GNB, Gram-negative bacteria; GPB, Gram-positive bacteria; ICU, intensive care unit; HSCT, hematopoietic stem-cell transplantation; OR, odds ratio; OS, overall survival; ST, sequence type; WGS, whole-genome sequencing; HAI, Hospital-acquired infections Sources: [9-25]

Reference	Country	Study Design	Setting	Population Description	Sample Size	Type of Infection	Pathogen Identified	AMR Pattern	Outcomes
Al-Mulla et al., 2014 [9]	Qatar	Retrospective cohort	Tertiary pediatric hematology and oncology center	Pediatric cancer patients (age ≤15 years)	185 patients; 70 with bloodstream infections; 111 bacteremia episodes	BSI	Gram-positive: *Staphylococcus epidermidis* (n=26) *Staphylococcus hominis* (n=9) *Staphylococcus haemolyticus* (n=7) Gram-negative: *Klebsiella pneumoniae* (n=14) *Pseudomonas aeruginosa* (n=10) *Escherichia coli* (n=7)	MDR organisms in 33 (28.4%) cases	Infection-related fatality rate: 2.2% (4 deaths)
Cornejo-Juárez et al., 2016 [10]	Mexico	Prospective cohort	ICU at a cancer referral center	Patients admitted to ICU for ≥48 hours; with hematological malignancies (HM), others with solid tumors	157 patients (104 with solid tumors, 53 with HM)	HAI	*Escherichia coli* *Candida glabrata* *C. tropicalis* *C. parapsilosis C. albicans Aspergillus fumigatus A. flavus*	MDR in 38 patients (24%) 78% of *E. coli* were ESBL producers	30-day mortality was 39%
Ilmi et al., 2022 [11]	Indonesia	Retrospective cross-Sectional	Pediatric hemato-oncology ward of Dr. Soetomo General Hospital, Surabaya	Pediatric hemato-oncology inpatients	Specimens n=196; positive cultures n=41	Mixed infections (blood, pus, urine)	Coagulase-negative *Staphylococcus* (50%); *E. coli* (40%); *K. pneumoniae* (30%); *P. aeruginosa* (15%)	MDR isolates: 80%; ESBL producers: 40%; AmpC and MDR *Pseudomonas* reported	-
Dhingra et al., 2020 [12]	India	Retrospective cohort	Hemato-oncology center in Tertiary Health Care Facility	Pediatric oncology patients with suspected episodes of septicemia	Blood cultures n=1423; positive cultures n=285	BSI	Coagulase-negative *Staphylococcus* (most common GPC); *Escherichia coli* (most common GNB)	Increasing carbapenem resistance among GNB; persistently high fluoroquinolone resistance; no resistance to glycopeptides or colistin	Mortality unchanged across periods (2.2% vs 2.6%)
Mvalo et al., 2018 [13]	South Africa	Retrospective cross-sectional	Pediatric oncology patients	Oncology patients	Positive cultures n=343; BSI episodes n=150 (89 patients)	BSI	Gram-positive bacteria (49.1%); Gram-negative bacteria (41.6%); fungi (9.3%). Common GP: CoNS, *Streptococcus viridans*; common GN: *E. coli*, *Klebsiella* spp.	Among *Enterobacteriaceae*, resistance was high to cephalosporins: cefepime 48.9%, ceftazidime 53.7%, and ceftriaxone 50%. Carbapenem resistance among *Enterobacteriaceae* was low: ertapenem 2.2%, imipenem 0%, and meropenem 2.3%. Among non-fermenting Gram-negative bacteria, imipenem resistance was 23.1% and meropenem resistance was 16.7%. For Gram-positive isolates, vancomycin resistance was 5.6%. The study reported two MRSA isolates and 14 ESBL-producing bacterial isolates.	Complications in 14% of BSI; case-fatality rate 2% (3 deaths)
Conn et al., 2017 [14]	Australia	Retrospective cohort	Tertiary referral hospital	Adults with acute leukemia or aggressive lymphoma receiving intensive chemotherapy	Patients n=212; blood cultures n=2324	BSI	Gram-negative bacteria (predominant); Gram-positive bacteria; anaerobes; *Candida tropicalis*	Resistance to first-line empiric therapy: 11.6%; ciprofloxacin resistance: 5%	30-day mortality following confirmed BSI
Gedik et al., 2014 [15]	Turkey	Retrospective cohort	Hematology department of a tertiary hospital	Patients ≥14 years with febrile neutropenia during chemotherapy for hematologic malignancies	Febrile episodes n=282 (126 patients)	Bacterial and fungal BSI	Gram-negative bacteria (74%), Gram-positive bacteria, and fungi	Carbapenem-resistant Gram-negative bacteria in 12% of GN bacteremia episodes	Mortality and treatment response assessed
Waker et al., 2020 [16]	Poland	Retrospective observational cohort	Single hemato-oncology ward	Patients with hematological malignancies	Tool samples n=2077; CDI-positive n=618; isolates sequenced n=140	Clostridium difficile infection	Clostridium difficile	Universal fluoroquinolone resistance; resistance to erythromycin (72.9%), clindamycin (42.9%), and moxifloxacin (72.9%); preserved susceptibility to metronidazole and vancomycin	-
Bastug et al., 2015 [17]	Turkey	Retrospective cohort	Tertiary-care teaching hospital	Patients with solid tumors or hematological malignancies	Patients n=132; infection episodes n=205	Sterile site infections	*Enterobacteriaceae* (44.3%) and nonfermentative isolates (17.6%)	MDR infections	-
Castagnola et al., 2021 [18]	Italy	Retrospective cohort (multicenter)	Multicenter study (14 centers)	Pediatric patients receiving chemotherapy or HSCT	Patients n=447; BSI episodes n=566	Gram-positive (60.9%); Gram-negative (39.1%). Common pathogens: *E. coli*, *S. aureus*, *K. pneumoniae*, *Streptococcus viridans*, *P. aeruginosa*	Resistance to ≥1 antibiotic: 49.9%; 3rd-gen cephalosporin resistance: *E. coli* 29.4%, *Klebsiella* spp. 60%; carbapenem resistance in *P. aeruginosa* 17.6%	Resistance to at least one antibiotic was observed in 49.9% of BSI. Resistance to third-generation cephalosporins in *E. coli* was 29.4%, and in *Klebsiella* spp. was 60%. Resistance to carbapenems in *P. aeruginosa* was 17.6%	Resistance associated with ICU admission (OR 2.43, 95% CI 1.44–4.11) and mortality (OR 3.19, 95% CI 1.45–7.01)
Uemura et al., 2017 [19]	Japan	Prospective observational cohort	Medical oncology and immunology care unit	Patients with hematological malignancies	Patients n=46	ESBL-producing *Enterobacteriaceae* outbreak	ESBL-producing *Enterobacteriaceae*	Marked increase in ESBL-producing isolates during outbreak period	Not specified
Leung et al., 2024 [20]	Hong Kong	Retrospective cohort	Pediatric intensive care unit	Critically ill children with hemato-oncology diagnoses or post-HSCT	PICU admissions n=124	ICU-associated infections	Bacterial, viral, and fungal infections	Antibiotic-resistant strains accounted for 61.3% of bacterial infections	Infections in 36.3% of admissions; bloodstream infection most common
Arman et al., 2022 [21]	Palestine	Cross-sectional study	Tertiary hematology center	Patients with hematologic malignancies and culture-positive infections	Isolates n=144	Culture-confirmed infections	Gram-negative bacteria, Gram-positive bacteria, and fungi	MDRO prevalence: 51.5% among GNB and 68.4% among GPB; difficult-to-treat resistance (DTR) in 16.7% of isolates	-
Sengar et al., 2014 [22]	India	Cross-sectional study	Tertiary care cancer center	Patients with hematological cancers (non-transplant)	Isolates n=739 (from 3,998 samples)	Bacterial infections	Predominantly Gram-negative bacteria (*E. coli*, *Klebsiella* spp., *Pseudomonas* spp.)	MDRO among GNB: 25.7%; ESBL producers: 41.4%; GN-MDRO among bloodstream isolates: 22%; MRSA: 1.3%; VRE: 1.3%	-
Mattei et al., 2022 [23]	Italy	Retrospective observational cohort	Tertiary-care pediatric hemato-oncology unit	Pediatric onco-hematological patients (0–18 years)	BSI episodes n=154 (111 patients)	BSI	Gram-positive bacteria, Gram-negative bacteria, and fungi	MDR among Gram-negative organisms: 18.8%; glycopeptide resistance among GP bacteria: 1%	- 30-day mortality was 7.1%. - Bacteremia-attributable mortality was 3.9%
Bajpai et al., 2023 [24]	India	Cohort study	Tertiary oncology center	Adults with hematolymphoid malignancies and febrile neutropenia.	Patients n=307	Bloodstream infections	Predominantly Gram-negative bacteria (*E. coli*, *K. pneumoniae*, *Pseudomonas* spp., *A. baumannii*)	MDR Gram-negative: 78.2%; carbapenem-resistant Gram-negative: 38.2%; MRSA: 15.8%; VRE: 5.3%	Overall mortality: 32.6%; MDR-BSI mortality: 42.5% vs. 11.2% in non-MDR BSI.
Scheich et al., 2018 [25]	Germany	Retrospective cohort	University Hospital Frankfurt	Patients with hematological malignancies	Patients n=109	BSI	Gram-negative bacteria	MDR gram-negative bacteria	Overall survival (OS) 30 days after BSI

**Table 2:** Regional stratification of the included studies.

Region	Included Countries/Studies	Main Findings
Asia	India, Qatar, Indonesia, Turkey, Japan, Hong Kong, Palestine	Asian studies contributed the largest proportion of evidence. Gram-negative organisms, ESBL-producing *Enterobacterales*, carbapenem-resistant Gram-negative organisms, and multidrug-resistant organisms were frequently reported, particularly in high-burden tertiary-care and hemato-oncology settings.
Europe	Poland, Italy, Germany	European studies showed heterogeneous resistance patterns. Pediatric multicenter data reported resistance to at least one antibiotic in a substantial proportion of bloodstream infections, while other studies reported organism-specific resistance patterns, including *Clostridioides difficile* antimicrobial resistance and multidrug-resistant Gram-negative bloodstream infections.
North America	None	No eligible North American study was included in the final synthesis.
Africa	South Africa	The South African pediatric oncology study reported bloodstream infections predominantly caused by Gram-positive bacteria, with low carbapenem resistance among *Enterobacteriaceae* and low overall case-fatality.
Latin America	Mexico	The Mexican ICU-based cancer cohort reported multidrug-resistant bacteria and a high proportion of ESBL-producing Escherichia coli, with substantial 30-day mortality.
Oceania/Australia	Australia	The Australian study reported bloodstream infections in adults receiving intensive chemotherapy for acute leukemia or aggressive lymphoma, with resistance to first-line empiric therapy documented in a minority of confirmed bloodstream infections.

Underlying populations included patients with acute leukemias and lymphoma and those receiving high-dose chemotherapy, immunotherapy, or radiotherapy or undergoing HSCT. Neutropenia was the dominant predisposing condition. Sample sizes ranged from 46 to 2,077 clinical samples. Common underlying malignancies included acute leukemias, lymphomas, and post-HSCT statuses. Neutropenia was the most frequently reported predisposing factor. Across all studies, blood cultures were the predominant clinical specimens assessed, followed by urine, respiratory samples, stool, and other sterile-site cultures. GNB and GPC were the principal pathogens isolated, with fungal organisms reported less frequently.

The included studies variably reported resistance at the patient level, infection episode level, or isolate level. Where possible, the original reporting unit was retained. Resistance percentages are therefore interpreted at the study level rather than pooled across uniform denominators.

Regional and Income-Setting Representation

The included studies were stratified by geographic region and World Bank income classification. Asia contributed the largest number of studies, followed by Europe. Africa, Latin America, and Oceania/Australia were represented by one study each, while no eligible North American study was included. Income-setting representation was mixed: eight studies were from high-income settings, and nine studies were from low- and middle-income countries. No study was from a low-income country (Table 3).

**Table 3:** Regional and income-setting stratification of the included studies. Income-setting summary: High-income countries contributed eight studies. Low- and middle-income countries contributed nine studies, including upper-middle-income and lower-middle-income settings. No included study was from a low-income country.

Region	Countries Represented	Number of Studies	Reported Sample Contribution by Original Study Denominator	World Bank Income Representation
Asia	Qatar, Indonesia, India, Turkey, Japan, Hong Kong, Palestine	10	920 patients/admissions where patient-level data were reported; 598 infection or bacteremia episodes; 5,617 specimens/cultures; 883 isolates; 326 positive cultures	High-income: Qatar, Japan, Hong Kong; upper-middle-income: Indonesia, Turkey; lower-middle-income: India, Palestine
Europe	Poland, Italy, Germany	4	667 patients where patient-level data were reported; 720 bloodstream infection episodes; 2,077 stool samples; 618 Clostridioides difficile-positive samples; 140 sequenced isolates	High-income: Poland, Italy, Germany
North America	None	0	No eligible study included	Not applicable
Africa	South Africa	1	89 patients; 150 bloodstream infection episodes; 343 positive cultures; 173 microbiological isolates	Upper-middle-income: South Africa
Latin America	Mexico	1	157 ICU patients overall, including 53 patients with hematologic malignancies	Upper-middle-income: Mexico
Oceania/Australia	Australia	1	212 patients; 2,324 blood cultures	High-income: Australia

Interpretation of regional sample contribution required caution because the included studies used heterogeneous denominator units. Some studies reported patient-level data, whereas others reported infection episodes, isolates, blood cultures, stool samples, or positive cultures. Therefore, regional contribution was summarized using the original denominator units reported by each study rather than by calculating a single pooled sample size.

Prevalence of antimicrobial resistance

Overall Resistance Burden

**Figure 2:**
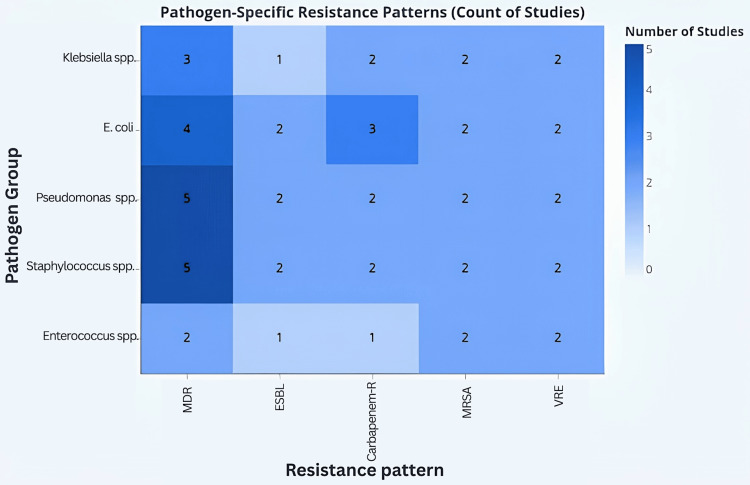
The heatmap illustrates the number of studies reporting each resistance phenotype (MDR, ESBL, carbapenem-resistant, MRSA, VRE) among major pathogen groups isolated from hemato-oncology patients. Darker shading represents higher frequency, indicating pathogens most consistently associated with resistant phenotypes across the evidence base. MDR, multidrug-resistant; MDRO, multidrug-resistant organism; ESBL, extended-spectrum β-lactamase; MRSA, methicillin-resistant *Staphylococcus aureus*; VRE, vancomycin-resistant *Enterococcus*

The most frequently reported resistance pattern is general multidrug resistance. Nine studies included in this synthesis explicitly mention the detection or concern of MDR organisms, and only one study directly reported that MDR was associated with mortality. The prevalence of MDR reported across the studies shows a wide range, spanning from 12% to 80% (Table 4, Figure 2).

**Table 4:** Study-level antimicrobial resistance patterns reported across the included hemato-oncology infection studies. This table presents only antimicrobial resistance values that were directly presented in the included studies and traceable to a reported organism group, resistance phenotype, study source, and denominator. Values are not pooled estimates. Because included studies used heterogeneous denominator units, including patients, infection episodes, bloodstream infection episodes, isolates, tested isolates, cultures, and specimens, resistance values should be interpreted within the context of the original study only and should not be compared as equivalent prevalence estimates across studies. AMR, antimicrobial resistance; BSI, bloodstream infection; CDI, *Clostridioides difficile* infection; CLSI, Clinical and Laboratory Standards Institute; CoNS, coagulase-negative staphylococci; CRE, carbapenem-resistant *Enterobacterales*; ESBL, extended-spectrum beta-lactamase; EUCAST, European Committee on Antimicrobial Susceptibility Testing; GNB, Gram-negative bacteria; GPB, Gram-positive bacteria; HSCT, hematopoietic stem-cell transplantation; ICU, intensive care unit; MDR, multidrug-resistant; MDRO, multidrug-resistant organism; MRSA, methicillin-resistant *Staphylococcus aureus*; VRE, vancomycin-resistant Enterococcus; XDR, extensively drug-resistant Sources: [10,13,18,20,24]

Organism/Pathogen Group	Resistance Phenotype	Study-Level Finding	Contributing Study/Studies	Denominator Type
Enterobacteriaceae	Cephalosporin resistance	Cefepime 48.9%, ceftazidime 53.7%, ceftriaxone 50%	Mvalo et al., 2018 [13]	Tested isolates
Enterobacteriaceae	Carbapenem resistance	Ertapenem 2.2%, imipenem 0%, meropenem 2.3%	Mvalo et al., 2018 [13]	Tested isolates
Non-fermenting Gram-negative bacteria	Carbapenem resistance	Imipenem 23.1%, meropenem 16.7%	Mvalo et al., 2018 [13]	Tested isolates
Gram-positive bacteria	Vancomycin resistance	5.60%	Mvalo et al., 2018 [13]	Tested isolates
Gram-negative bacteria	MDR	78.2%; carbapenem-resistant GNB 38.2%	Bajpai et al., 2023 [24]	Study-defined clinical cohort
Bacterial infections	Antibiotic-resistant strains	61.3% of bacterial infections	Leung et al., 2024 [20]	Bacterial infections
Bloodstream infections	Resistance to ≥1 antibiotic	49.90%	Castagnola et al., 2021 [18]	BSI episodes/isolates as reported
Escherichia coli	ESBL	78% of *E. coli* were ESBL producers	Cornejo-Juárez et al., 2016 [10]	Isolates/patients as reported

Gram-Negative Resistance

Gram-negative bacteria constituted the predominant pathogen group and demonstrated the highest burden of resistance in hemato-oncology patients. ESBL-producing organisms were frequently identified among *Escherichia coli *and *Klebsiella pneumoniae *isolates. Carbapenem resistance was widespread, particularly within *Enterobacterales, Pseudomonas aeruginosa,* and *Acinetobacter baumannii*. Fluoroquinolone resistance exceeded 40-60% in most reported cohorts, especially in centers employing quinolone prophylaxis and aminoglycoside resistance was common among *Pseudomonas* isolates. Colistin susceptibility was generally retained, although isolated reports of emerging colistin resistance were noted.

Gram-Positive Resistance

Methicillin-resistant *Staphylococcus aureus* (MRSA) constituted up to 50% of *S. aureus* isolates in some studies. Moreover, coagulase-negative staphylococci (CoNS) frequently showed multidrug-resistant profiles. Resistance to macrolides and clindamycin was variably reported, whereas susceptibility to vancomycin and linezolid generally remained high.

Fungal Resistance

Fungal pathogens were reported in a minority of included studies, but antifungal susceptibility testing was inconsistently reported. Therefore, antifungal resistance was not formally synthesized.

Multidrug-Resistant Organisms

MDR and MDRO estimates varied substantially across the included studies. These estimates should be interpreted cautiously because the studies used different denominator units and MDR definitions. Some studies reported MDR at the patient level, others at the infection episode level, and others at the isolate level. Therefore, MDR percentages were not treated as directly comparable prevalence estimates.

Where isolate-level or organism-level MDRO data were available, high-burden cohorts reported substantial MDR proportions, particularly among Gram-negative organisms. However, these values were not pooled because studies differed in organism grouping, susceptibility-testing standards, resistance definitions, geographic setting, infection type, and denominator structure. MDR was interpreted according to the definitions used by the original studies, generally corresponding to resistance to at least one agent in three or more antimicrobial classes, but the criteria were not uniform across all studies.

Overall, MDR burden appeared highest among Gram-negative pathogens, particularly *Enterobacterales*, *Pseudomonas aeruginosa*, and *Acinetobacter baumannii* in selected high-burden settings. However, because the available data were reported using heterogeneous denominators and non-uniform MDR definitions, the findings are presented as descriptive study-level observations rather than pooled prevalence estimates. No single overall MDR prevalence estimate was calculated.

Microorganisms With the Highest Resistance Rates

The pathogens consistently exhibiting the highest AMR rates across studies were (1) *Klebsiella pneumoniae* (ESBL, CRE); (2) *Pseudomonas aeruginosa* (carbapenem- and aminoglycoside-resistant); (3) *Acinetobacter baumannii* (MDR and XDR phenotypes); (4) ESBL-producing *Escherichia coli*; and (5) MRSA and MDR CoNS (in catheter-related infections). These organisms were also disproportionately implicated in severe infections, septic shock, and mortality.

Geographical and Temporal Trends

Geographical variation was one of the most important sources of heterogeneity in the included studies. Asia contributed the largest number of studies, but the evidence was not evenly distributed across Asian subregions. South Asia was mainly represented by Indian cohorts, while additional studies came from Qatar, Indonesia, Turkey, Japan, Hong Kong, and Palestine. Europe was represented by Poland, Italy, and Germany, while Africa, Latin America, and Oceania/Australia were each represented by one study. No eligible study from North America was included. Therefore, the findings should be interpreted as regionally heterogeneous study-level observations rather than as a single global estimate.

The resistance burden appeared higher in selected Asian and Middle Eastern cohorts than in several European or Australian cohorts included in this review. Indian studies reported substantial Gram-negative resistance, including multidrug-resistant Gram-negative bacteria and carbapenem-resistant Gram-negative bacteria. Bajpai et al. reported MDR Gram-negative bacteria in 78.2% and carbapenem-resistant Gram-negative bacteria in 38.2% of cases in an adult febrile neutropenia cohort [24]. Sengar et al. reported MDROs among 25.7% of Gram-negative bacteria and ESBL producers in 41.4% [22]. Arman et al. from Palestine reported MDRO prevalence of 51.5% among Gram-negative bacteria and 68.4% among Gram-positive bacteria, with difficult-to-treat resistance in 16.7% of isolates [21]. These findings suggest that high-resistance settings may have a particularly heavy burden of resistant Gram-negative infections in hemato-oncology populations.

In contrast, some cohorts from Europe, Australia, and South Africa reported lower or more selective resistance burdens. Conn et al. from Australia reported resistance to first-line empiric therapy in 11.6% and ciprofloxacin resistance in 5% of confirmed bloodstream infections [14]. Mvalo et al. from South Africa reported low carbapenem resistance among *Enterobacteriaceae*, including ertapenem resistance of 2.2%, imipenem resistance of 0%, and meropenem resistance of 2.3%, although resistance among non-fermenting Gram-negative bacteria was higher [13]. European studies showed heterogeneous findings: Castagnola et al. reported resistance to at least one antibiotic in 49.9% of bloodstream infections, third-generation cephalosporin resistance in 29.4% of *Escherichia coli* and 60% of *Klebsiella* spp., and carbapenem resistance in 17.6% of *Pseudomonas aeruginosa* [18], while Mattei et al. reported MDR among Gram-negative organisms in 18.8% [23].

Temporal interpretation was limited because the included studies were not designed as continuous surveillance studies and did not use uniform denominators or susceptibility definitions. However, when studies were considered descriptively by publication period, earlier studies from 2014-2018 documented important baseline resistance signals, including ESBL-producing *Enterobacterales*, MDR Gram-negative bacteria, carbapenem-resistant Gram-negative bacteria, MRSA, VRE, and resistant *Clostridioides difficile* isolates. Later studies from 2019-2025 more frequently reported high-burden MDR phenotypes, carbapenem-resistant Gram-negative bacteria, difficult-to-treat resistance, and outcome associations with resistant bloodstream infection.

These temporal patterns should not be interpreted as proof of a formal upward trend because no included study provided long-term, standardized, multicenter surveillance suitable for time-series analysis. Rather, the available evidence suggests persistent and regionally variable expansion of clinically important resistance phenotypes, particularly MDR and carbapenem-resistant Gram-negative organisms, in hemato-oncology settings.

Clinical outcomes related to AMR

Mortality

Mortality outcomes were reported heterogeneously across included studies and were therefore summarized according to the original outcome definition used by each study. Mortality estimates were not pooled because studies differed in follow-up period, denominator unit, attribution method, patient population, infection type, and adjustment for disease severity.

Infection-related fatality was reported by Al-Mulla et al., who described an infection-related fatality rate of 2.2% [9]. Mvalo et al. reported a bloodstream infection case-fatality rate of 2%, with three deaths occurring during the bloodstream infection [13]. Mattei et al. reported 30-day mortality of 7.1% and bacteremia-attributable mortality of 3.9% [23]. Cornejo-Juárez et al. reported 30-day mortality of 39% in an intensive care unit-based cancer cohort [10]. Bajpai et al. reported an overall mortality of 32.6%, with mortality higher among patients with MDR BSI than among those with non-MDR BSI [24]. Castagnola et al. reported an association between resistant bloodstream infection and mortality, with an odds ratio of 3.19 (95% CI 1.45-7.01) [18]. Studies that did not report extractable mortality outcomes were not included in the mortality summary (Table 5).

**Table 5:** Mortality outcomes reported across the included studies according to outcome definition. MDR, multidrug-resistant; BSI, bloodstream infection

Outcome Definition	Study	Reported Estimate	Denominator/Context	Interpretation
Infection-related fatality	Al-Mulla et al., 2014	2.20%	Pediatric hematology/oncology bloodstream infection cohort	Infection-attributed fatality; not directly comparable with all-cause mortality
Case-fatality during BSI	Mvalo et al., 2018	2.00%	3 deaths among 150 BSI episodes	Case-fatality during bloodstream infection episodes
30-day mortality	Mattei et al., 2022	7.10%	Pediatric onco-hematology BSI episodes	Time-defined mortality after bloodstream infection
Bacteremia-attributable mortality	Mattei et al., 2022	3.90%	Pediatric onco-hematology BSI episodes	Mortality attributed specifically to bacteremia
30-day mortality	Cornejo-Juárez et al., 2016	39.00%	ICU-based cancer cohort including hematologic malignancy patients	ICU-based mortality; high-risk setting
Overall mortality	Bajpai et al., 2023	32.60%	Adults with hematolymphoid malignancy and febrile neutropenia-associated BSI	Overall mortality, not limited to infection-attributed death
MDR-BSI versus non-MDR-BSI mortality	Bajpai et al., 2023	42.5% vs. 11.2%	MDR bloodstream infection compared with non-MDR bloodstream infection	Comparative study-level mortality finding
Mortality association	Castagnola et al., 2021	OR 3.19, 95% CI 1.45–7.01	Resistant bloodstream infection versus non-resistant bloodstream infection	Association estimate; not a prevalence value

These findings indicate that resistant infections were often associated with worse outcomes, but the magnitude of mortality risk could not be estimated across studies because mortality definitions and denominator units were not comparable.

Other Clinical Outcomes

Across studies, AMR infections were associated with (1) higher ICU admission rates, (2) prolonged hospitalization, (3) increased need for last-line therapies such as colistin or tigecycline, (4) delayed clinical response, (5) higher incidence of septic shock, and (6) increased recurrence or breakthrough bacteremia. These findings collectively indicate that AMR is a strong negative predictor of clinical outcomes in hemato-oncology populations.

Resistance to Last-Resort Antibiotics

Multiple studies documented rising resistance to carbapenems among *Enterobacterales* and non-fermenters. ESBL-producing organisms were frequently reported among *Enterobacterales*, but reporting varied by organism and denominator. Explicit ESBL-related values included ESBL producers in 40% in Ilmi et al. [11], ESBL producers among 41.4% of Gram-negative organisms in Sengar et al. [22], and ESBL-producing *E. coli* in 78% of *E. coli* isolates in Cornejo-Juárez et al. [10]. Mvalo et al. [13] reported 14 ESBL-producing bacterial isolates. Uemura et al. [19] described an outbreak-related increase in ESBL-producing *Enterobacteriaceae* but did not provide a pooled prevalence suitable for combination with other studies. Some studies reported third-generation cephalosporin resistance rather than ESBL production; these were not treated as identical. Castagnola et al. [18] reported third-generation cephalosporin resistance in 29.4% of *E. coli* and 60% of *Klebsiella* spp. bloodstream infection isolates, while Mvalo et al. [13] reported cefepime resistance of 48.9%, ceftazidime resistance of 53.7%, and ceftriaxone resistance of 50% among tested *Enterobacteriaceae* isolates.

Carbapenem resistance was reported sparsely. Mvalo et al. [13] reported low carbapenem resistance among *Enterobacteriaceae*, with ertapenem resistance of 1/45 (2.2%), imipenem resistance of 0/43 (0%), and meropenem resistance of 1/44 (2.3%). In contrast, carbapenem resistance among non-fermenting Gram-negative bacteria was higher in the same study, with imipenem resistance of 3/13 (23.1%) and meropenem resistance of 2/12 (16.7%). Gedik et al. [15] reported carbapenem-resistant Gram-negative bacteria in 12% of Gram-negative bacteremia episodes. Castagnola et al. [18] reported carbapenem resistance in 17.6% of *P. aeruginosa* bloodstream infection isolates. Bajpai et al. [24] reported carbapenem-resistant Gram-negative bacteria in 38.2% of cases in an adult febrile neutropenia cohort. These findings show substantial study-level variation, including higher carbapenem-resistance burden in selected non-European cohorts, but the estimates were not pooled because denominators and organism groupings differed.

Research gaps

The current synthesis identified multiple substantive gaps in the evidence base on AMR in hemato-oncology, spanning epidemiology, microbiology, surveillance practices, treatment strategies, and reporting standards. These gaps limit the ability to derive regionally relevant empirical therapy recommendations, hinder early detection of resistant pathogens, and constrain the development of tailored stewardship interventions (Table 6).

**Table 6:** Research gaps and corresponding future research directions in AMR among hemato-oncology patients. AMR, antimicrobial resistance; MDR, multidrug-resistant; XDR, extensively drug-resistant; CLSI, Clinical and Laboratory Standards Institute; EUCAST, European Committee on Antimicrobial Susceptibility Testing; LMICs, low- and middle-income countries; HSCT, hematopoietic stem-cell transplantation; CAR-T, chimeric antigen receptor T-cell

Research Gap Identified	Detail	Recommended Future Research Direction
1. Limited high-quality prospective studies	Evidence is mostly from a single-center retrospective cohort with poor temporal resolution.	Develop multicenter, prospective surveillance cohorts with standardized protocols.
2. Heterogeneous AMR definitions & reporting	MDR/XDR definitions vary between studies, and breakpoints are not standardized (CLSI vs. EUCAST).	Adopt universal reporting standards; require clear definitions and susceptibility criteria.
3. Minimal genomic/molecular epidemiology	Few studies assess resistance genes or clonal outbreaks.	Integrate whole-genome sequencing, resistome profiling, and molecular outbreak tracking.
4. Insufficient data from LMICs	Highest AMR burden regions are underrepresented; surveillance fragmented.	Establish region-specific surveillance networks.
5. Underrepresentation of HSCT, CAR-T, and immunotherapy patients	These ultra-high-risk groups are poorly studied.	Conduct dedicated cohort studies for HSCT/CAR-T/immunotherapy recipients.
6. Poor characterization of antifungal resistance	Limited susceptibility testing	Expand antifungal resistance surveillance and analyze azole/echinocandin resistance mechanisms.

Discussion

This systematic review summarizes antimicrobial resistance patterns and associated clinical outcomes among patients with hematologic malignancies. Across the included studies, resistant Gram-negative organisms were frequently reported, particularly ESBL-producing *Enterobacterales*, carbapenem-resistant Gram-negative bacteria, and MDR Gram-negative organisms. Resistance to third-generation cephalosporins, fluoroquinolones, and carbapenems was reported in several cohorts, although estimates varied substantially by geographic region, organism group, infection type, susceptibility-testing standard, resistance definition, and denominator unit. Resistant infections were generally associated with poorer clinical outcomes in observational cohorts, including ICU admission, prolonged hospitalization, and mortality. However, these associations should not be interpreted as evidence of causality because most included studies were observational and subject to confounding.

The findings highlight the vulnerability of patients with hematologic malignancies to resistant infections, particularly during febrile neutropenia, intensive chemotherapy, HSCT, mucosal barrier injury, central venous catheter use, and repeated exposure to broad-spectrum antimicrobials. The available evidence suggests that resistant Gram-negative infections represent a major clinical concern in hemato-oncology settings. However, the present review did not include general hospitalized populations as a comparator group; therefore, it cannot quantify whether the antimicrobial resistance burden is higher than that of non-hemato-oncology populations. The findings should instead be interpreted as evidence that antimicrobial resistance is clinically important in this high-risk immunocompromised population.

The findings of this review are broadly consistent with global and regional antimicrobial resistance surveillance reports in identifying resistant Gram-negative bacteria as a major public-health concern. The WHO Global Antimicrobial Resistance Surveillance System report [26] and the ECDC European antimicrobial resistance surveillance report [27] provide a broader epidemiological context for resistance among major bacterial pathogens. These surveillance sources document the importance of resistance among *Enterobacterales* and non-fermenting Gram-negative organisms, including resistance to third-generation cephalosporins and carbapenems. However, these surveillance data are not specific to hematologic malignancy populations and should not be treated as direct comparators for the present review.

The present review also differs from geographically restricted reviews. Baccelli et al. reviewed resistant bacterial infections in hematologic malignancy and hematopoietic cell transplant populations within Europe and reported a median carbapenem-resistance estimate of approximately 13% [28]. Our findings are directionally consistent with that review in identifying resistant Gram-negative pathogens as clinically important in hemato-oncology populations. However, direct comparison of resistance magnitude is limited because the present review included broader geographic regions, including South Asia, the Middle East, Africa, Latin America, Europe, and Australia/Oceania.

Several studies in the present review reported carbapenem-resistance burdens higher than those described in the Europe-focused review. For example, Bajpai et al. reported carbapenem-resistant Gram-negative bacteria in 38.2% of cases in an Indian adult febrile neutropenia cohort [24]. These higher values should not be interpreted as conflicting with Baccelli et al. [28]. Rather, they likely reflect expected geographic variation in antimicrobial-resistance ecology, antimicrobial exposure, infection-control infrastructure, microbiology surveillance capacity, patient risk profile, and local empirical treatment practices. Therefore, the present findings support the need for region-specific antimicrobial surveillance and empirical therapy guidance rather than extrapolation from Europe-only estimates.

Lalaoui et al. focused specifically on carbapenem-resistant infections in adult patients with hematologic malignancies and identified high mortality associated with carbapenem-resistant Enterobacterales, particularly in patients with acute leukemia and persistent neutropenia [29]. Colonization with carbapenem-resistant organisms was also highlighted as an important risk factor for subsequent infection. Our broader synthesis is consistent with that review in showing that carbapenem resistance is clinically important in hemato-oncology settings. However, because the included evidence is predominantly observational, carbapenem resistance should be described as being associated with adverse outcomes rather than as a proven causal driver of morbidity or mortality.

Several methodological factors may have influenced the reported antimicrobial resistance rates. Included studies used different antimicrobial susceptibility interpretation standards, including CLSI and EUCAST criteria. Differences in breakpoint criteria can affect whether isolates are classified as susceptible, intermediate, or resistant, particularly for Gram-negative organisms and last-line agents. In addition, MDR was not defined uniformly. Some studies used formal definitions based on resistance to at least one agent in three or more antimicrobial classes, whereas others reported broader categories such as MDROs, ESBL-producing *Enterobacterales*, carbapenem-resistant Gram-negative bacteria, difficult-to-treat resistance, or resistance to first-line empirical therapy. These categories overlap clinically but are not interchangeable epidemiological measures. Furthermore, microbiological surveillance practices differed across centers. Studies based only on clinically indicated cultures may preferentially capture more severe infections and may miss colonization or subclinical acquisition of resistant organisms. In contrast, centers using routine surveillance cultures, screening protocols, or more intensive microbiological sampling may detect a higher burden of resistant organisms. Differences in culture frequency, specimen type, laboratory capacity, and reporting thresholds may therefore influence apparent resistance prevalence.

BSI cohorts and mixed infection cohorts are not directly comparable. BSI studies generally represent microbiologically confirmed invasive infections and often include more severely ill patients, whereas mixed infection cohorts may include urinary, respiratory, gastrointestinal, catheter-related, skin and soft tissue, sterile-site, or *Clostridioides difficile* infections, depending on study design. These differences affect pathogen distribution, denominator type, clinical severity, and measured outcomes. For this reason, resistance estimates in the present review were summarized descriptively at the study level rather than pooled.

The findings of this review have practical implications for hemato-oncology units, particularly in settings with high burdens of MDR Gram-negative organisms. Colonization screening may be considered in high-risk units where ESBL-producing *Enterobacterales*, carbapenem-resistant *Enterobacterales*, MDR *Pseudomonas aeruginosa*, or *Acinetobacter* species are prevalent. Rectal or stool surveillance cultures may help identify patients colonized with resistant Gram-negative organisms before invasive infection develops. Screening strategies should be adapted to local epidemiology, laboratory capacity, infection-control resources, and antimicrobial stewardship priorities.

Empirical antibiotic therapy for febrile neutropenia should be risk-adapted rather than uniform across all settings. Relevant patient-level and unit-level factors include prior colonization or infection with resistant organisms, recent broad-spectrum antimicrobial exposure, previous carbapenem use, duration and depth of neutropenia, HSCT status, mucositis, central venous catheter use, hemodynamic instability, ICU admission, and local resistance patterns. In high-resistance settings, standard empirical regimens may require modification to ensure early active therapy, while unnecessary escalation should be avoided in lower-risk patients to reduce further selective pressure.

Local antibiogram data should be integrated into hemato-oncology treatment algorithms. Unit-specific antibiograms may be more informative than hospital-wide antibiograms because hemato-oncology patients often have different antimicrobial exposure histories, pathogen distributions, and resistance risks. Regular review of local bloodstream infection isolates, ESBL prevalence, carbapenem resistance, fluoroquinolone resistance, aminoglycoside susceptibility, colistin susceptibility, and newer-agent susceptibility may support more rational empirical therapy and de-escalation decisions.

Newer agents may have a role in selected high-resistance settings. Ceftazidime-avibactam, meropenem-vaborbactam, imipenem-cilastatin-relebactam, and cefiderocol may be relevant for infections caused by carbapenem-resistant Gram-negative organisms, depending on organism identification, resistance mechanism, susceptibility testing, drug availability, infection site, severity of illness, and expert consultation. These agents should not be used indiscriminately or as routine empirical therapy for all febrile neutropenia episodes. Instead, they should be incorporated into antimicrobial stewardship pathways for patients with known colonization, previous infection with resistant organisms, septic shock, or local epidemiology indicating high risk of inactive standard empirical therapy.

While this review provides a focused synthesis of antimicrobial resistance patterns in hemato-oncology populations, several methodological considerations should be acknowledged. Substantial clinical, microbiological, methodological, and epidemiological heterogeneity existed across included studies. Studies differed in age group, hematologic diagnosis, transplant status, chemotherapy exposure, infection syndrome, pathogen grouping, antimicrobial susceptibility-testing standards, resistance definitions, and outcome reporting. This heterogeneity precluded quantitative meta-analysis and limited direct comparability of resistance rates. Most included studies were observational, and many were retrospective. Therefore, associations between antimicrobial resistance and adverse clinical outcomes cannot establish causality. Patients with resistant infections may also differ from those with susceptible infections in severity of underlying malignancy, neutropenia duration, prior antimicrobial exposure, HSCT status, ICU admission, catheter exposure, mucosal barrier injury, and baseline clinical instability. Residual confounding could not be excluded. Denominator units varied substantially across studies. Some studies reported patient-level data, others episode-level data, and others isolate-level or tested-isolate-level susceptibility data. Therefore, resistance percentages were interpreted according to the denominator used in the original study and were not treated as equivalent prevalence estimates.

Geographic representation was uneven. Asia contributed the largest number of studies, while Africa, Latin America, and Oceania/Australia were represented by one study each, and no eligible North American study was included. As a result, the findings may not fully represent global hemato-oncology antimicrobial resistance patterns. Temporal trend analysis was not feasible. The included studies were not designed as continuous surveillance studies and did not use uniform methods across time. Therefore, apparent temporal differences should be interpreted descriptively and should not be considered evidence of a formal time trend. Fungal resistance was incompletely captured. Fungal pathogens were reported in some studies, but antifungal susceptibility testing was inconsistent and fungal-only resistance studies were not the primary focus of this review. Therefore, antifungal resistance could not be formally synthesized. The original PROSPERO registration indicated that quantitative meta-analysis would be considered where appropriate; however, meta-analysis was not performed because the included studies were too heterogeneous for valid pooling. This deviation from the pre-registered synthesis plan was handled by performing a descriptive study-level synthesis.

The evidence gaps identified in this review point toward several priorities for future research. Prospective multicenter surveillance studies are needed to generate reliable region-specific estimates of antimicrobial resistance in hematologic malignancy populations. Future studies should collect standardized patient-level, infection episode-level, isolate-level, and outcome-level data. Standardized antimicrobial resistance reporting is also needed. Future studies should report organism-specific resistance phenotypes, including ESBL production, carbapenem resistance, multidrug resistance, MRSA, VRE, fluoroquinolone resistance, aminoglycoside resistance, colistin resistance, and antifungal resistance where relevant. Reports should include clear numerator-denominator values, denominator type, susceptibility-testing standards, and resistance definitions. Interventional studies should evaluate hemato-oncology-specific antimicrobial stewardship strategies. These studies should assess risk-adapted empirical therapy, early de-escalation, prophylaxis policies, colonization-guided treatment algorithms, and integration of local antibiogram data into febrile neutropenia pathways.

Future studies should also evaluate rapid molecular diagnostics and resistance prediction models. Rapid organism identification, molecular resistance testing, whole-genome sequencing, machine-learning-based risk prediction, and electronic health record-integrated decision support may help identify patients at high risk for resistant infection and guide earlier effective therapy while supporting antimicrobial stewardship.

## Conclusions

Antimicrobial resistance is a clinically important challenge in patients with hematologic malignancies, particularly due to multidrug-resistant Gram-negative pathogens. Resistant infections were associated with poorer outcomes in observational cohorts, but causality cannot be inferred. Strengthened hemato-oncology-specific surveillance, local antibiogram-guided empirical therapy, antimicrobial stewardship, and standardized AMR reporting are needed to improve infection management in this high-risk population.

## Appendices

Information sources and search strategy

1. The final PubMed search string was: 

("Hematologic Neoplasms"[MeSH] OR "hematologic malignancy" OR "haematological cancer" OR leukemia OR lymphoma OR "multiple myeloma")

AND

("Drug Resistance, Microbial"[MeSH] OR "antimicrobial resistance" OR "multidrug resistant" OR MDR OR ESBL OR "extended spectrum beta lactamase" OR "carbapenem resistant" OR CRE OR MRSA OR VRE)

AND

("Bloodstream Infection"[MeSH] OR bacteremia OR sepsis OR infection)

2. Scopus search strategy; The PubMed search was adapted for Scopus syntax as follows:

2.1.  TITLE-ABS-KEY ("hematologic malignancy" OR "haematological cancer" OR leukemia OR lymphoma OR "multiple myeloma")

AND

TITLE-ABS-KEY ("antimicrobial resistance" OR "multidrug resistant" OR MDR OR ESBL OR "carbapenem resistant" OR CRE OR MRSA OR VRE)

AND

TITLE-ABS-KEY (infection OR bacteremia OR sepsis)

2.2. TITLE-ABS-KEY ("hematologic malignancy" OR "haematological malignancy" OR "hematologic cancer" OR "haematological cancer" OR leukemia OR leukaemia OR lymphoma OR "multiple myeloma") AND TITLE-ABS-KEY ("antimicrobial resistance" OR "antibiotic resistance" OR "multidrug resistant" OR MDR OR MDRO OR ESBL OR "extended-spectrum beta-lactamase" OR "carbapenem resistant" OR "carbapenem-resistant" OR CRE OR MRSA OR VRE) AND TITLE-ABS-KEY (infection OR bacteremia OR bacteraemia OR sepsis OR "febrile neutropenia")

3. Google scholar search strategy 

Google Scholar was searched as a supplementary database to identify additional studies not indexed in PubMed or Scopus and recently published articles not yet fully indexed. The search was restricted to the first 200 relevance-ranked results.

Google Scholar search combination; “hematologic malignancy,” “haematological malignancy,” “leukemia,” “lymphoma,” “multiple myeloma,” “antimicrobial resistance,” “antibiotic resistance,” “multidrug resistant,” “ESBL,” “carbapenem resistant,” “MRSA,” “VRE,” “bloodstream infection,” “bacteremia,” “sepsis,” and “febrile neutropenia.”

Risk of bias interpretation

Based on Newcastle-Ottawa Scale (NOS) scoring, studies were categorized as low risk (≥7 points), moderate risk (5-6 points), or high risk (<5 points).

**Table 7:** Quality assessment using the Newcastle-Ottawa Scale for cohort studies [7]. Sources: [9,10,12,14-20,23-25]

Reference	Selection (Max score-4)				Comparability (2)	Outcome (Max 3)			Total Score
	Representativeness of Cohort	Selection of Non-exposed Cohort	Ascertainment of Exposure	Outcome Not Present at Start	Comparability	Assessment of Outcome	Follow-up Long Enough	Adequacy of Follow-up	
Al-Mulla et al., 2014 [9]	1	0	1	1	0	1	1	1	6
Cornejo-Juárez et al., 2016 [10]	1	1	1	1	1	1	1	0	7
Dhingra et al., 2020 [12]	1	0	0	1	2	1	1	1	7
Conn et al., 2017 [14]	1	0	1	1	0	1	1	0	5
Gedik et al., 2014 [15]	1	0	1	1	0	1	1	1	6
Waker et al., 2020 [16]	1	0	0	1	0	1	1	1	5
Bastug et al., 2015 [17]	1	0	0	1	0	1	1	1	5
Castagnola et al., 2021 [18]	1	0	1	0	2	1	1	1	7
Uemura et al., 2017 [19]	1	0	0	1	1	1	1	0	5
Leung et al., 2024 [20]	1	0	1	1	2	1	1	0	7
Mattei et al., 2022 [23]	1	0	1	1	2	1	1	1	8
Bajpai et al., 2023 [24]	1	0	1	1	0	1	1	1	6
Scheich et al., 2018 [25]	1	0	1	1	0	1	1	0	5

**Table 8:** Quality assessment using the Newcastle-Ottawa Scale for cross-sectional studies [7]. Sources: [11,13,21,22]

Reference	Selection (Max score-5)					Comparability (2)	Outcome (Max 3)			Grand Total [10]
	Representativeness of Sample	Sample Size	Non-respondents	Ascertainment of Exposure	Ascertainment of Outcome	Confounder Control	Assessment of Outcome	Statistical Test	Data Completeness	
Ilmi et al., 2022 [11]	1	1	1	1	1	1	1	0	1	8
Mvalo et al., 2018 [13]	1	1	1	1	1	1	1	0	0	7
Arman et al., 2022 [21]	1	1	1	1	1	1	1	0	1	8
Sengar et al., 2014 [22]	1	1	1	1	1	0	1	1	1	8

## References

[1] Averbuch D, Sureda A, Corbacioglu S (2024). Bacterial infections. The EBMT Handbook.

[2] Salam MA, Al-Amin MY, Salam MT (2023). Antimicrobial resistance: a growing serious threat for global public health. Healthcare (Basel).

[3] Hosseini MS, Jahanshahlou F, Akbarzadeh MA (2024). Formulating research questions for evidence-based studies. J Med Surg Public Health.

[4] Sona PH, Attavar PC, Rasmi TR (2024). Emergence of high-level antibiotic resistance in Klebsiella pneumoniae: a narrative review. South Asian J Res Microbiol.

[5] Acebo JJ, Bhattacharyya P, Escobedo-Melendez G (2025). Infections in immunosuppressed pediatric patients. Pediatric Surgical Oncology.

[6] Page MJ, McKenzie JE, Bossuyt PM (2021). The PRISMA 2020 statement: an updated guideline for reporting systematic reviews. BMJ.

[7] Saiprashanth L, V P A, Shreya B (2025). Antimicrobial resistance patterns in hemato-oncology. Open Science Framework.

[8] Carra MC, Romandini P, Romandini M (2025). Risk of bias evaluation of cross-sectional studies: adaptation of the Newcastle-Ottawa scale. J Periodontal Res.

[9] Al-Mulla NA, Taj-Aldeen SJ, El Shafie S, Janahi M, Al-Nasser AA, Chandra P (2014). Bacterial bloodstream infections and antimicrobial susceptibility pattern in pediatric hematology/oncology patients after anticancer chemotherapy. Infect Drug Resist.

[10] Cornejo-Juárez P, Vilar-Compte D, García-Horton A, López-Velázquez M, Ñamendys-Silva S, Volkow-Fernández P (2016). Hospital-acquired infections at an oncological intensive care cancer unit: differences between solid and hematological cancer patients. BMC Infect Dis.

[11] Ilmi UNA, Sri Rejeki IG, Ugrasena DG (2022). Antibiotic susceptibility pattern of Gram-positive bacteria in the pediatric hemato-oncology ward of Dr. Soetomo General Hospital, Surabaya, Indonesia. Biochem Cell Arch.

[12] Dhingra H, Kalra M, Mendiratta L (2018). Fighting the rising spectre of tough bugs: a ten year experience from a tertiary care centre in North India. Pediatr Hematol Oncol J.

[13] Mvalo T, Eley B, Bamford C (2018). Bloodstream infections in oncology patients at Red Cross War Memorial Children's Hospital, Cape Town, from 2012 to 2014. Int J Infect Dis.

[14] Conn JR, Catchpoole EM, Runnegar N, Mapp SJ, Markey KA (2017). Low rates of antibiotic resistance and infectious mortality in a cohort of high-risk hematology patients: a single center, retrospective analysis of blood stream infection. PLoS One.

[15] Gedik H, Simşek F, Kantürk A (2014). Bloodstream infections in patients with hematological malignancies: which is more fatal - cancer or resistant pathogens?. Ther Clin Risk Manag.

[16] Waker E, Ambrozkiewicz F, Kulecka M (2020). High prevalence of genetically related Clostridium difficile strains at a single hemato-oncology ward over 10 years. Front Microbiol.

[17] Bastug A, Kayaaslan B, Kazancioglu S (2015). Emergence of multidrug resistant isolates and mortality predictors in patients with solid tumors or hematological malignancies. J Infect Dev Ctries.

[18] Castagnola E, Bagnasco F, Mesini A (2021). Antibiotic resistant bloodstream infections in pediatric patients receiving chemotherapy or hematopoietic stem cell transplant: factors associated with development of resistance, intensive care admission and mortality. Antibiotics (Basel).

[19] Uemura M, Imataki O, Uchida S (2017). Strain-specific transmission in an outbreak of ESBL-producing Enterobacteriaceae in the hemato-oncology care unit: a cohort study. BMC Infect Dis.

[20] Leung KK, Ho PL, Wong SC, Chan WY, Hon KL (2025). Prevalence and outcomes of infections in critically-ill paediatric oncology patients: a retrospective observation study. Curr Pediatr Rev.

[21] Arman G, Zeyad M, Qindah B (2022). Frequency of microbial isolates and pattern of antimicrobial resistance in patients with hematological malignancies: a cross-sectional study from Palestine. BMC Infect Dis.

[22] Sengar M, Kelkar R, Jain H, Biswas S, Pawaskar P, Karpe A (2014). Frequency of bacterial isolates and pattern of antimicrobial resistance in patients with hematological malignancies: a snapshot from tertiary cancer center. Indian J Cancer.

[23] Mattei D, Baretta V, Mazzariol A (2022). Characteristics and outcomes of bloodstream infections in a tertiary-care pediatric hematology-oncology unit: a 10-year study. J Clin Med.

[24] Bajpai Vijeta, Kumar Amit, Mandal Tanmoy (2023). Prevalence of multidrug resistant bloodstream infections in febrile neutropenic patients with hematolymphoid malignancies: a retrospective observational study from a newly established tertiary oncology center in India. Cancer Res Stat Treat.

[25] Scheich S, Weber S, Reinheimer C (2018). Bloodstream infections with gram-negative organisms and the impact of multidrug resistance in patients with hematological malignancies. Ann Hematol.

[26] (2026). Global antibiotic resistance surveillance report 2025: summary. report.

[27] (2026). Antimicrobial resistance surveillance in Europe 2022 - 2020 data. Europe.

[28] Baccelli F, Aguilar-Guisado M, Vidal CG (2025). Epidemiology of resistant bacterial infections in patients with hematological malignancies or undergoing hematopoietic cell transplantation in Europe: a systematic review by the European Conference on Infections in Leukemia (ECIL). J Infect.

[29] Lalaoui R, Javelle E, Bakour S, Ubeda C, Rolain JM (2020). Infections due to carbapenem-resistant bacteria in patients with hematologic malignancies. Front Microbiol.

